# The relationship between emotional states and mental well-being in men: mediating effect of leisure-time physical activity

**DOI:** 10.3389/fpsyg.2026.1794057

**Published:** 2026-04-01

**Authors:** Cemal Güler, Cemre Can Akkaya, Anıl Siyahtaş, Tarkan Havadar, Nurgül Kaya, Cemile Nihal Yurtseven, Ataman Tükenmez, Bülent Duran

**Affiliations:** 1 Faculty of Sports Sciences, Istanbul University-Cerrahpaşa, Istanbul, Türkiye; 2 Faculty of Sports Sciences, Istanbul Yeni Yuzyil University, Istanbul, Türkiye; 3 Turkish Football Federation, Istanbul, Türkiye; 4 Sarıkamış Faculty of Sports Sciences, Kafkas University, Kars, Türkiye

**Keywords:** anxiety, depression, leisure-time physical activities, mental well-being, stress

## Abstract

**Background:**

There is evidence that emotional states such as depression, anxiety, and stress negatively affect individuals’ mental well-being. The present study aims to examine the potential protective effect of leisure-time physical activity (LTPA) in men and to provide empirical data on the roles of this variable in psychological health.

**Method:**

The study group for this cross-sectional research consisted of 270 males with high physical activity level, recruited from fitness centers, with a mean age of 27.29 (±10.46) years. A three-variable mediation analysis was conducted in this research. The bootstrap method was applied to the analyses, and the statistical significance of the mediation effect was evaluated using 95% bootstrap confidence intervals.

**Results:**

The results indicated that LTPA significantly mediated the relationships between depression and mental well-being (*β* = −0.020; 95% CI [−0.046, −0.005]) and between anxiety and mental well-being (*β* = −0.023; 95% CI [−0.052, −0.005]). However, the indirect effect of LTPA on the relationship between stress and mental well-being was not statistically significant (95% CI [−0.041, 0.018]).

**Conclusion:**

In conclusion, LTPA showed a significant statistical mediating association in the relationship between depression, anxiety, and mental well-being; however, no significant mediating association was observed in the relationship involving stress. These findings indicate that LTPA offers a protective mechanism, particularly for mood disorders (depression and anxiety), but that additional interventions may be needed for stress management.

## Introduction

Leisure-time physical activity (LTPA) is defined as any bodily movement produced by skeletal muscles that requires energy expenditure. Physical activity encompasses all movements performed during leisure time, while moving for transportation, or during activities related to an individual’s work and domestic life (World Health Organization, 2024). Previous research has comprehensively reported the health-related benefits of leisure-time physical activity. LTPA can lead to a significant reduction in the incidence of dementia and the risk of mortality (Sun et al., 2023). Additionally, higher levels of physical activity during leisure time are associated with a reduced risk of cardiovascular disease (Wu et al., 2024). While LTPA can be one of the ways to reduce musculoskeletal morbidity (Hildebrandt et al., 2000), it may also have significant and protective effects on bone mineral density in postmenopausal women (Siyahtaş et al., 2026). Furthermore, it also provides significant physical health benefits, including the improvement of body composition (Musijowska and Kwilosz, 2024), the prevention of overweight and obesity (Petermann-Rocha et al., 2025), the reduction of chronic musculoskeletal pain (Baday-Keskin and Keskin, 2025), and the reduction of the risk of metabolic syndrome (He et al., 2014). The health benefits of leisure-time physical activities are not limited solely to the physiological dimension.

The protective effects of a lifestyle that includes leisure-time physical activity are well known, and there are various methods to encourage people to be active so that they can enjoy better physical and mental well-being and a higher quality of life. Leisure-time physical activity triggers specific physiological and biochemical changes in the brain and body, and also creates changes in perceptions and experiences related to the environment and one’s own body, which helps improve psychological functioning (Trajković et al., 2023). The positive effect of physical activity is largely attributed to the release of endorphins, which help improve mood and increase the activity of the prefrontal cortex, which is responsible for emotional regulation and decision-making processes (Al-Wardat et al., 2024). Despite these findings, the psychological benefits of regular physical activity and the psychological factors that may influence this activity are less clear. Therefore, studies investigating the link between physical activity and psychological health have become increasingly important in recent years (Doré et al., 2019; Gaia et al., 2022; Eather et al., 2023).

Leisure-time physical activity stands out as a powerful method for improving mental health, promoting emotional balance, and coping with stress (Siyahtaş et al., 2025). Physical activity not only improves mood but also alleviates symptoms of disorders such as depression and Anxiety (Martín-Rodríguez et al., 2024; Noetel et al., 2024). While leisure-time physical activities can provide protective effects against depressive symptoms (Sabiston et al., 2016), they can also help mitigate anxiety symptoms (Ashdown-Franks et al., 2017). Physical activity offers a method for coping with stress and can help people regulate their emotions by enabling them to understand and control them (Al-Wardat et al., 2024). Therefore, considering the multi-faceted benefits physical activity offers to individuals, it is imperative that this behavior be adopted as a sustainable (Liu et al., 2024). The numerous benefits provided by LTPA are considered to be of particular importance for men’s health. This is because men are less likely than women to seek help for psychological problems (Bye et al., 2025). This situation is described as an easily accessible intervention tool for men to cope with emotional problems without the risk of stigmatization (Currier et al., 2020). Furthermore, the high prevalence of inactivity among men highlights the importance of promoting leisure-time physical activity in this population. Therefore, the present research aims to examine the relationships between emotional states, mental health, and leisure physical activity in men.

The relationship between physical activity and emotional states and mental health has been documented in considerable detail, but the underlying mechanisms of these relationships are not sufficiently clear. The extent to which physical activity functions as a consistent explanatory mechanism across various emotional domains has not been systematically investigated. Therefore, it remains unclear whether leisure-time physical activity has a consistent effect on the links between different negative emotional states and mental health, or whether its psychological impact varies with specific emotional states. Addressing this issue, this study focuses on the same male sample and examines the mediating role of leisure-time physical activity in the relationships between depression, anxiety, stress, and mental well-being. By comparing these emotional constructs using a consistent analytical method, the research attempts to determine whether leisure-time physical activity functions consistently or situationally across these conditions. Thus, this study offers a mechanism-based and broader perspective beyond unidirectional relationships to research on physical activity and psychological health in men.

## Literature review

Mental well-being, as a core component of psychological health, refers to an individual’s emotional well-being and appropriate behavioral adjustment. This construct encompasses relative freedom from debilitating symptoms, such as anxiety, and the ability to establish constructive relationships. Furthermore, it also incorporates the capacity to cope with the normal demands and stresses of life (Gautam et al., 2024). Research indicates that mental well-being is shaped by the interaction of various intrinsic and environmental factors (Ryff and Singer, 2013). Among the wide range of factors influencing mental well-being, emotional states such as depression, anxiety, and stress are considered among the most critical determinants. These factors have been consistently associated with impairments in emotional regulation, cognitive functioning, and social adjustment. In particular, prolonged exposure to stress and elevated levels of anxiety and depressive symptoms may undermine individuals’ psychological resilience and overall mental health. Consequently, depression, anxiety, and stress are frequently emphasized as central risk factors in mental health research (Alvi et al., 2022).

Depression is a significant emotional disorder prevalent in all age groups, negatively affecting both individuals’ productivity and social relationships. It may manifest with symptoms such as persistent sadness, lack of motivation, feelings of worthlessness, and pessimism, and often impairs daily functioning. Due to its high risk of recurrence, depression is regarded not only as an individual concern but also as a public health problem. Therefore, it is important to implement preventive and supportive measures to mitigate its negative effects and preclude its development in healthy individuals (Yüceant, 2022). In addition to depression, anxiety and stress are also defined as significant mental disorders that negatively affect individuals’ mental health. Research shows that increased levels of stress and anxiety are inversely proportional to people’s psychological health (Naganandini, 2024; Yüksel and Bahadir-Yilmaz, 2019). Studies conducted on different groups reveal that symptoms of stress, anxiety, and depression are often experienced together and that these conditions show strong positive correlations with each other (Dülger and Ayaz-Alkaya, 2025). This data proves that these three psychological disorders have significant and negative effects on individuals’ mental health (Salari et al., 2020). Based on the explanations above, the following hypotheses are proposed in the current study:

*H1a*: There is a relationship between depression and mental well-being.

*H1b*: There is a relationship between anxiety and mental well-being.

*H1c*: There is a relationship between stress and mental well-being.

Physical activities are among the most popular activities during leisure (Zorba and Yermakhanov, 2022). Although physical activity may not possess an effect as potent as traditional antidepressants, it can offer ameliorative contributions in a similar direction toward reducing depressive symptoms (Mahindru et al., 2023). In addition to pharmacological methods, regular participation in leisure-time physical activity can serve as an effective complementary approach with preventive and therapeutic potential for various adverse health conditions (Bendau et al., 2024). Leisure-time physical activity can provide a marked and long-lasting reduction in the severity of depressive symptoms (Noetel et al., 2024). Physical activities promote the restoration of neurotransmitter balances in the brain, support hormonal regulation, and reduce inflammation levels. These biological effects contribute to a decrease in depression risk by positively influencing individuals’ mental state (Dishman et al., 2006; Kandola et al., 2019). The reduction in depression leads to a significant improvement in individuals’ mental health levels and facilitates the strengthening of mental well-being (Yüceant, 2022).

Previous studies have provided evidence that anxiety influences individuals’ levels of mental well-being (Pacesova et al., 2018; Kwon and Cho, 2020). Therefore, LTPA can be an important supportive factor in managing emotional disorders such as anxiety (Kandola and Stubbs, 2020). Physical activity affects the underlying biological and psychological mechanisms of anxiety in a multi-dimensional way, and is regarded as a complementary and prospective intervention area in the clinical management of anxiety disorders (Anderson and Shivakumar, 2013). Furthermore, research also supports this notion (Lin and Gao, 2023). The physical activity has been found to contribute independently to greater well-being and lower levels of anxiety and depressive symptoms (McMahon et al., 2017).

Stress is regarded as a significant health problem at both the individual and societal levels in modern societies (Doney et al., 2022). Physical activity reduces the physiological arousal caused by stress, supports the relaxation response, and strengthens individuals’ stress coping skills (Salmon, 2001). Furthermore, physical activity increases the release of endorphins in the brain, positively influencing mood balance and contributing to the reduction of stress levels in the long term (Dinas et al., 2011). With the reduction of stress, positive effects on mental well-being are observed, and individuals’ levels of mental well-being increase (Salmon, 2001). Consequently, the LTPA is regarded as a feasible and sustainable intervention area in the non-pharmacological management of stress (Mikkelsen et al., 2017). Although the current literature demonstrates the negative effects of emotional states on mental well-being, this relationship appears to have been largely understudied in the context of non-pharmacological interventions. Therefore, the following hypotheses has been developed:

*H2a*: LTPA mediates the relationship between depression and mental well-being.

*H2b*: LTPA mediates the relationship between anxiety and mental well-being.

*H2c*: LTPA mediates the relationship between stress and mental well-being.

## Method

### Research model

**Figure 1:**
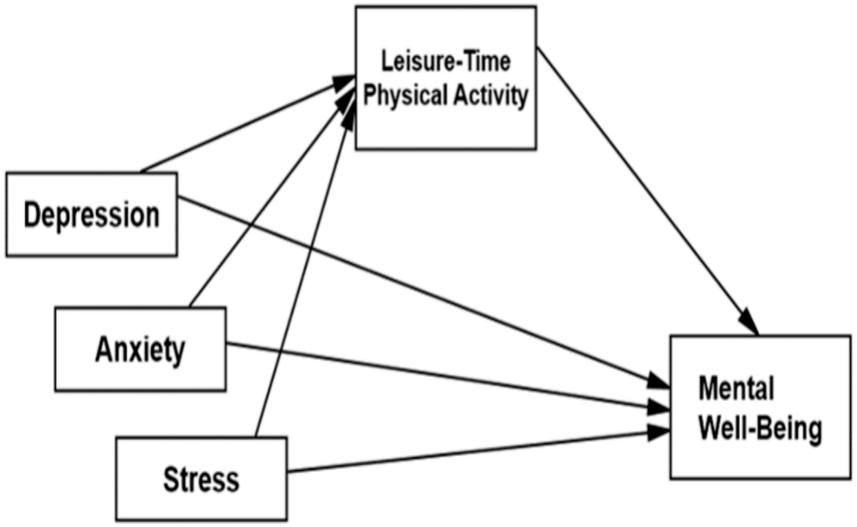
Research model.

The main objective of this cross-sectional study is to investigate the mediating role of leisure-time physical activity in the relationship between emotional states and mental well-being in men. Accordingly, depression, anxiety, and stress (as emotional states) were identified as independent variables, while mental well-being was identified as the dependent variable. The mediating role of leisure-time physical activity in the relationship between these two variables was tested in the model. Direct and indirect relationships between the variables were analyzed using path analysis conducted in AMOS. Since the proposed mediation model was specified using observed variables, the analysis represents a path model rather than a full latent variable structural equation model. The conceptualized model is presented in Figure 1.

### Research group

The study group of this research is comprised of individuals participating in leisure-time physical activity in fitness centers located in a district of Istanbul Province. The research group was determined using convenience sampling to increase accessibility. During the data collection process, a face-to-face questionnaire method was applied to the participants. The inclusion criteria for the research were determined as follows: being a Turkish citizen, being male, and being within the age range of 18–45 years. Within this framework, data were collected from a total of 282 participants; however, 12 participants identified as having incomplete questionnaires were excluded from the scope of the study. The exclusion criteria for the research were not being a Turkish citizen, being under 18 or over 45 years of age, and filling out the questionnaire form incompletely or incorrectly. The analyses were performed using valid data obtained from 270 participants (*n* = 270) who met the criteria.

A power analysis was conducted using the G*Power 3.1 software to evaluate the adequacy of the sample size utilized in the research. It is stated that in studies where the effect size is considered medium (Cohen, 1988), the minimum required sample size for a 95% confidence level and 80% statistical power ranges approximately between 150 and 200 participants. The results of the power analysis determined that the research met the minimum required sample size for the planned statistical tests.

### Data collection tools

The data collection instruments used in the research were the Personal Information Form prepared by the researchers, the International Physical Activity Questionnaire-Short Form (IPAQ-SF), the Depression, Anxiety, and Stress Scale-21 (DASS-21), and the Mental Well-being Scale (MWBS). The personal information form has been prepared to collect information about participants’ age, educational status, income level, and marital status.

The leisure-time physical activity levels of the male participants were analyzed using the International Physical Activity Questionnaire-Short Form (IPAQ-SF). The seven-item questionnaire assesses individuals’ walking, moderate-intensity activity, vigorous-intensity activity, and sitting time during the last 7 days. Data obtained from the questionnaire are calculated using MET-minute/week values and classified into low, moderate, and high physical activity levels. Physical activity levels were divided into three categories based on the total MET-minute/week value: Low physical activity level was classified as ≤ 600 MET-min/week, moderate physical activity level as 601–3.000 MET-min/week, and high physical activity level as > 3.000 MET-min/week. In this study, leisure-time physical activity was assessed based on participants’ physical activity levels over the past 7 days using the IPAQ-SF. The measurement does not directly assess the habit strength or automaticity dimension of the behavior. It reflects self-reported physical activity volume. Therefore, within the scope of this study, the physical activity variable indicates current physical activity participation levels rather than psychological habit formation.

The Depression, Anxiety, and Stress Scale-21 (DASS-21) was used to evaluate participants’ depression, anxiety, and stress levels. The scale was developed by Lovibond and Lovibond (1995) and adapted into Turkish by Sarıçam (2018). The scale consists of 21 items and measures individuals’ depression, anxiety, and stress levels over the last week using a 4-point Likert-type format. The DASS-21’s internal consistency coefficients were determined as 0.87 for the depression subscale, 0.85 for the anxiety subscale, and 0.81 for the stress subscale. The scale classifies symptom severity into five categories: normal, mild, moderate, severe, and extremely severe. For the depression subscale, scores between 0–4 are classified as normal, 5–6 as mild, 7–10 as moderate, 11–13 as severe, and 14 and above as extremely severe. For anxiety, scores of 0–3 indicate normal levels, 4–5 mild, 6–7 moderate, 8–9 severe, and 10 and above extremely severe. For stress, scores ranging from 0–7 are categorized as normal, 8–9 as mild, 10–12 as moderate, 13–16 as severe, and 17 and above as extremely severe. These classification thresholds were used to interpret participants’ psychological symptom levels in the present study (Sarıçam, 2018). As a result of the reliability analyses calculated for this study, the Cronbach’s alpha coefficients were determined as *α* = 0.854 for depression, α = 0.894 for anxiety, and α = 0.880 for stress, respectively.

The Warwick-Edinburgh Mental Well-being Scale (MWBS) was used to assess participants’ levels of mental well-being. The scale was developed by Tennant et al. (2007) and adapted into Turkish by Keldal (2015). The scale consists of 14 items, each assessed on a 5-point Likert-type scale. Total scores range from 14 to 70, and increasing scores indicate a high level of mental well-being. The scale’s internal consistency coefficient was determined to be 0.89 (Keldal, 2015). In the reliability analysis conducted in the current study, the overall Cronbach’s alpha coefficient was calculated as α = 0.917.

### Procedure

Ethics approval to conduct the research was unanimously obtained from the Istanbul University-Cerrahpaşa Social and Humanities Research Ethics Committee during its meeting no. 500 on 05.08.2025. Participants were informed about the general purpose of the research and were asked to participate voluntarily. Informed consent was obtained from the participants before participating in the study, and the entire process was carried out in accordance with the Declaration of Helsinki and ethical rules.

### Data analysis

Data obtained from the research were analyzed using SPSS 25.0 (Statistical Package for the Social Sciences) and AMOS 25.0 software. First, descriptive statistics (mean, standard deviation, frequency, and percentage) were calculated to examine the participants’ demographic characteristics and the distribution of the main variables. Since the data showed skewness and kurtosis values ranging between −1.5 and +1.5, the distribution was concluded to be normal (Tabachnick and Fidell, 2013). In correlational analyses, the correlations between the variables were evaluated using the Pearson correlation test. A three-variable mediation analysis was performed in the research. In this analysis, the independent variable, the dependent variable, and the mediating variable were identified, and the direct and indirect effects between the variables were examined. The analyses were repeated with 2.000 bootstrap samples using the bootstrap method to assess the reliability of the indirect effect. Based on the bootstrap confidence intervals (95% CI), it was determined whether the mediation effect was statistically significant. The fact that the confidence interval of the indirect effect does not include zero indicates that the mediation is significant. The mediation model was tested using path analysis conducted in AMOS with observed variables. As the specified model was fully saturated (df = 0), global fit indices (e.g., CFI, TLI, RMSEA, SRMR) were not reported.

## Results

In Table 1, the demographic characteristics of the participants are presented. The mean age of the participants was 27.29 ± 10.46 years, and 73.7% of them were single. Regarding income status, the majority of the participants (70.4%) were in the middle-income group. In terms of educational level, high school graduates constituted the largest proportion with 44.4%.

**Table 1:** Demographic characteristics of participants.

Variable	Groups	f	%	x̄±sd
Age				27.29 ± 10.46
Marital status	Married	71	26.3	
	Single	199	73.7	
Income level	Low	45	16.7	
	Moderate	190	70.4	
	High	35	13.0	
Educational status	Primary school	7	2.6	
	Middle school	47	17.4	
	High school	120	44.4	
	University	96	35.6	
Total		270	100.0	

Table 2 presents the mean scores of the participants and the relationships between leisure-time physical activity level and psychological variables. The participants’ physical activity level was determined as an average of 5134.81 MET-min/week. The mean depression score was 5.71(±5.02), the mean anxiety score was 5.19(±5.07), the mean stress score was 6.13(±5.08), and the mean mental well-being score was 53.49(±10.85). According to the correlation results, there was a negative and significant relationship between LTPA and depression (*r* = −0.150, *p* < 0.05), as well as between LTPA and anxiety (*r* = −0.174, *p* < 0.01). Additionally, LTPA was positively and significantly associated with mental well-being (*r* = 0.210, *p* < 0.01). Mental well-being showed moderate negative correlations with depression (*r* = −0.551, *p* < 0.01), anxiety (*r* = −0.478, *p* < 0.01), and stress levels (*r* = −0.502, *p* < 0.01).

**Table 2:** Scale score means and correlation results.

Variables	Mean- MET-min/Week	Sd	1	2	3	4
1. Leisure physical activity	5134.81					
2. Depression	5.71	5.02	−0.150*			
3. Anxiety	5.19	5.07	−0.174**	0.812**		
4. Stress	6.13	5.08	−0.05	0.812**	0.815**	
5. Mental well-being	53.49	10.85	0.210**	−0.551**	−0.478**	−0.502**

*p* < 0.05*; *p* < 0.01**.

The results regarding the mediating role of LTPA in the relationship between depression and mental well-being are presented in Table 3. According to the table, depression was negatively and significantly associated with mental well-being (*β* = −0.532; Bootstrap 95% CI [−0.623, −0.429]; Percentile 95% CI [−0.623, −0.430]).

**Table 3:** The mediating role of LTPA in the relationship between depression and mental well-being.

Direction of effect	Path relationship	Estimate	Boot SE	Bootstrap 95% CI		Percentile 95% CI	
				LLCI	ULCI	LLCI	ULCI
Direct effect	D—MWB	−0.532	0.049	−0.623	−0.429	−0.623	−0.430
Indirect effect	D—LTPA—MWB	−0.020	0.010	−0.046	−0.005	−0.044	−0.004
Total effect	D—LTPA—MWB	−0.551	0.048	−0.638	−0.446	−0.640	−0.450

D, Depression; LTPA, Leisure-Time Physical Activity; MWB, Mental Well-being.

The analysis of indirect effects indicated a negative and statistically significant indirect association between depression and mental well-being through LTPA (*β* = −0.020; Bootstrap 95% CI [−0.046, −0.005]; Percentile 95% CI [−0.044, −0.004]).

Regarding the total association, the findings indicated that depression was negatively and significantly associated with mental well-being (*β* = −0.551; Bootstrap 95% CI [−0.638, −0.446]; Percentile 95% CI [−0.640, −0.450]).

The results regarding the mediating role of LTPA in the relationship between anxiety and mental well-being are presented in Table 4. According to the table, anxiety was negatively and significantly associated with mental well-being (*β* = −0.455; Bootstrap 95% CI [−0.562, −0.353]; Percentile 95% CI [−0.564, −0.353]).

**Table 4:** The mediating role of LTPA in the relationship between anxiety and mental well-being.

Direction of effect	Path relationship	Estimate	Boot SE	Bootstrap 95% CI		Percentile 95% CI	
				LLCI	ULCI	LLCI	ULCI
Direct effect	A—MWB	−0.455	0.053	−0.562	−0.353	−0.564	−0.353
Indirect effect	A—LTPA—MWB	−0.023	0.011	−0.052	−0.005	−0.049	−0.005
Total effect	A—LTPA—MWB	−0.478	0.051	−0.575	−0.377	−0.583	−0.380

A, Anxiety; LTPA, Leisure-Time Physical Activity; MWB, Mental Well-being.

The analysis of indirect effects indicated a negative and statistically significant indirect association between anxiety and mental well-being through LTPA (*β* = −0.023; Bootstrap 95% CI [−0.052, −0.005]; Percentile 95% CI [−0.049, −0.005]).

Regarding the total association, the findings indicated that anxiety was negatively and significantly associated with mental well-being (*β* = −0.478; Bootstrap 95% CI [−0.575, −0.377]; Percentile 95% CI [−0.583, −0.380]).

The results regarding the mediating role of LTPA in the relationship between stress and mental well-being are presented in Table 5. According to the table, stress was negatively and significantly associated with mental well-being (*β* = −0.492; Bootstrap 95% CI [−0.582, −0.397]; Percentile 95% CI [−0.581, −0.397]).

**Table 5:** The mediating role of LTPA in the relationship between stress and mental well-being.

Direction of effect	Path relationship	Estimate	Boot SE	Bootstrap 95% CI		Percentile 95% CI	
				LLCI	ULCI	LLCI	ULCI
Direct effect	S—MWB	−0.492	0.047	−0.582	−0.0397	−0.581	−0.397
Indirect effect	S—LTPA—MWB	−0.011	0.015	−0.041	0.018	−0.042	0.017
Total effect	S—LTPA—MWB	−0.502	0.047	−0.589	−0.408	−0.589	−0.408

S, Stress; LTPA, Leisure-Time Physical Activity; MWB, Mental Well-being.

The analysis of indirect effects indicated that the indirect association between stress and mental well-being through LTPA was not statistically significant (*β* = −0.011), as the 95% bootstrap confidence interval included zero (Bootstrap 95% CI [−0.041, 0.018]; Percentile 95% CI [−0.042, 0.017]).

Regarding the total association, the findings indicated that stress was negatively and significantly associated with mental well-being (*β* = −0.502; Bootstrap 95% CI [−0.589, −0.408]; Percentile 95% CI [−0.589, −0.408]).

## Discussion

The findings of this study indicate that males participating in leisure-time physical activity in fitness centers exhibit a high level of physical activity. Participants’ weekly activity levels fall into the high physical activity category, and previous research suggests that higher levels of physical activity are positively associated with both physical health and mental well-being. The current study found a negative correlation between emotional states (depression, anxiety, stress) and mental well-being. Consequently, hypothesis H1 developed in the research was accepted, and the results are also supported by the existing literature (Gürgan and Gür, 2019). This negative correlation shows that the intense psychological symptoms experienced by individuals lower their mental well-being (Maddock, 2024). In particular, the increase in stress and anxiety levels associated with depressive symptoms limits an individual’s daily functioning and undermines their mental resilience by depleting their internal resources. During this process, an increase in depression, anxiety, and stress levels can be a precursor to physical inactivity (Denche-Zamorano et al., 2022). This situation can create a cycle that further worsens mental well-being (Casanova et al., 2023). At this point, rather than focusing solely on clinical treatments to combat negative emotional states, it is necessary to also address lifestyle changes that affect individuals’ mental well-being (Amiri et al., 2024). It is emphasized that non-pharmacological methods such as physical activity stabilize mood by triggering the release of neurotransmitters such as serotonin and endorphins, and offer a low-cost and sustainable strategy that supports mental well-being and complements pharmacological treatments (Schuch and Vancampfort, 2021). The findings suggest that mental well-being interventions may benefit from not only addressing symptom reduction but also incorporating protective strategies aimed at enhancing mental well-being. Therefore, managing emotional states and making leisure-time physical activity can play a critical role in protecting an individual’s psychological integrity.

Previous studies have provided evidence of the positive effects of leisure-time physical activity on mental well-being (Feil et al., 2021; Peng et al., 2022; Liu et al., 2024). Therefore, Hypothesis 2a (H2a), proposing that LTPA is positively associated with mental well-being and may play a mediating role in the relationship between depression and mental well-being, was supported. According to the research findings, depression was negatively associated with mental well-being, and LTPA were found to play a mediating role in this relationship. In other words, the model indicated that a portion of the total association between depression and mental well-being (*β* = −0.551) was explained through the indirect pathway involving LTPA. This indicates that depression indirectly reduces mental well-being by weakening men’s physical activity. The results confirm that LTPA is a critical mediating variable that modifies the negative correlation between depression and mental well-being. Similar studies conducted support the findings of the present study (Zuo et al., 2025). LTPA is not merely a health behavior; it may function as a protective factor that helps reduce the psychological impact of depression in men. Therefore, LTPA should be considered an important health-promoting behavior. Regular participation in leisure-time physical activity may enhance psychological resilience by supporting adaptive coping processes.

Anxiety, like depression, negatively affects mental well-being (Loureiro et al., 2025). While current research findings confirm this relationship, it has been found that LTPA mediate this relationship. Therefore, the developed H2b has been accepted. According to the analysis results, when the total association between anxiety and mental well-being (β = −0.478) was examined, a significant portion of this association was attributable to the direct pathway within the model. However, it has been found that LTPA indirectly affect this relationship. Studies in the literature have shown consistency with the findings of the present study (Kekäläinen et al., 2020; Fuentealba-Urra et al., 2023; Rodríguez-Romo et al., 2023; Deng et al., 2024). Increased anxiety levels in men negatively affect leisure-time physical activity. This indirectly makes mental well-being even more fragile. The results support the theory that symptoms such as avoidance behavior caused by anxiety in men can disrupt physical activity. However, the significant mediating role of LTPA suggests that maintaining regular participation in leisure-time physical activity may serve as a supportive mechanism for preserving mental well-being among men experiencing anxiety.

The average physical activity level of the sample group in this study (5134.81 MET-min/week) significantly exceeds the threshold of 3,000 MET-min/week defined in the literature as ‘high physical activity’. This exceptional activity level indicates that the sample consists of individuals who are already highly engaged in leisure-time physical activity. This situation necessitates evaluating the mediating role of leisure-time physical activity in the context of this specific sample. The current findings reveal that even at such high MET levels, leisure-time physical activity continues to mediate the relationship between depression/anxiety and mental well-being. This finding indicates that even when the total amount of physical activity is very high, the protective effect on mental well-being persists when the activity is performed in a ‘leisure-time’ context.

In contrast to findings in depression and anxiety, this research has shown that leisure-time physical activity does not play a mediating role in the relationship between stress and mental well-being. Accordingly, H2c was rejected. This finding can be interpreted in terms of both the sample characteristics and the existing literature. Previous studies have provided evidence that physical activity may play a mediating role between stress and mental well-being. For example, Kwag et al. (2011) reported that physical activity significantly mediated the relationship between stress and psychological health indicators. However, in the current study, the inconsistency that emerged when this mediating role was replicated in the male sample group is thought to stem from the general characteristics of the participants. In this study, the average stress score of male participants (6.13) corresponds to a normal severity level according to the scale classification. This indicates that participants generally reported low stress levels. Theoretically, it can be said that the stress-reducing effects of physical activity are likely to be more pronounced in individuals experiencing high levels of psychological distress. Supporting this perspective, Hegberg and Tone (2015) found that the protective role of physical activity is more effective in individuals with high levels of psychological distress; in contrast, it did not show the same level of protective effect in individuals in the low or medium risk group. Therefore, the low stress levels observed in the current sample group, i.e., among men, may suggest that the stress levels observed in the current sample were not sufficiently elevated to reveal a potential buffering association. Furthermore, the limited variability in stress scores may have reduced the statistical capacity to detect indirect effects within the proposed mediating model. However, since no subgroup or threshold analysis was conducted to directly examine differences in stress levels, it is considered that these interpretations should be evaluated cautiously. Future research should retest the mediating role of leisure-time physical activity in sample groups with higher stress levels or more pronounced psychological distress to determine whether the finding becomes more pronounced. Additionally, stress is a multidimensional construct (e.g., perceived, chronic, situational), and different forms of stress may relate differently to physical activity. Future research incorporating differentiated stress measures may help clarify these potential associations.

### Limitations

The current study has some limitations. The characteristics of the study group are among the most significant limitations. Participants consist solely of male individuals. Participants selected from fitness centers are seen to have a high level of physical activity. This type of sampling method may create potential selection bias. The findings may be limited in their applicability to sedentary individuals or people from different sociocultural backgrounds, particularly female. Therefore, the results should be evaluated within the context of the physically active male population. Including more diverse participant groups in terms of gender, and sampling source in future studies is expected to increase external validity. In this study, the average physical activity level of the sample group (Mean = 5134.81 MET-min/week) is well above the threshold value accepted in the literature for high physical activity. This indicates that the research findings reflect a group of highly active individuals. Therefore, the mediated relationships obtained may not be directly generalizable to sedentary or moderately active individuals. Testing similar models on samples with different physical activity levels in future studies will contribute to a more robust assessment of the generalizability of the findings. Another limitation of the current study relates to its design. The study was conducted using a cross-sectional design. Although this design allows for the examination of associations between variables and the statistical testing of mediation models, it does not establish temporal ordering among the variables and therefore does not permit causal inferences. Accordingly, the mediation findings should be interpreted as statistical mediation rather than evidence of causal pathways. The results reflect relational patterns observed at a single point in time. Future research employing longitudinal or experimental designs would be necessary to clarify temporal sequencing and to more rigorously examine potential causal mechanisms. Another important limitation of this study is that it measured leisure-time physical activity using the IPAQ-SF, which assesses physical activity levels over the past 7 days, rather than a scale measuring the habit strength or behavioral automaticity dimension of physical activity. Therefore, the findings point to current activity participation levels rather than the psychological habit formation of physical activity. Future research should use tools that directly measure behavioral habit strength.

## Conclusion

**Figure 2:**
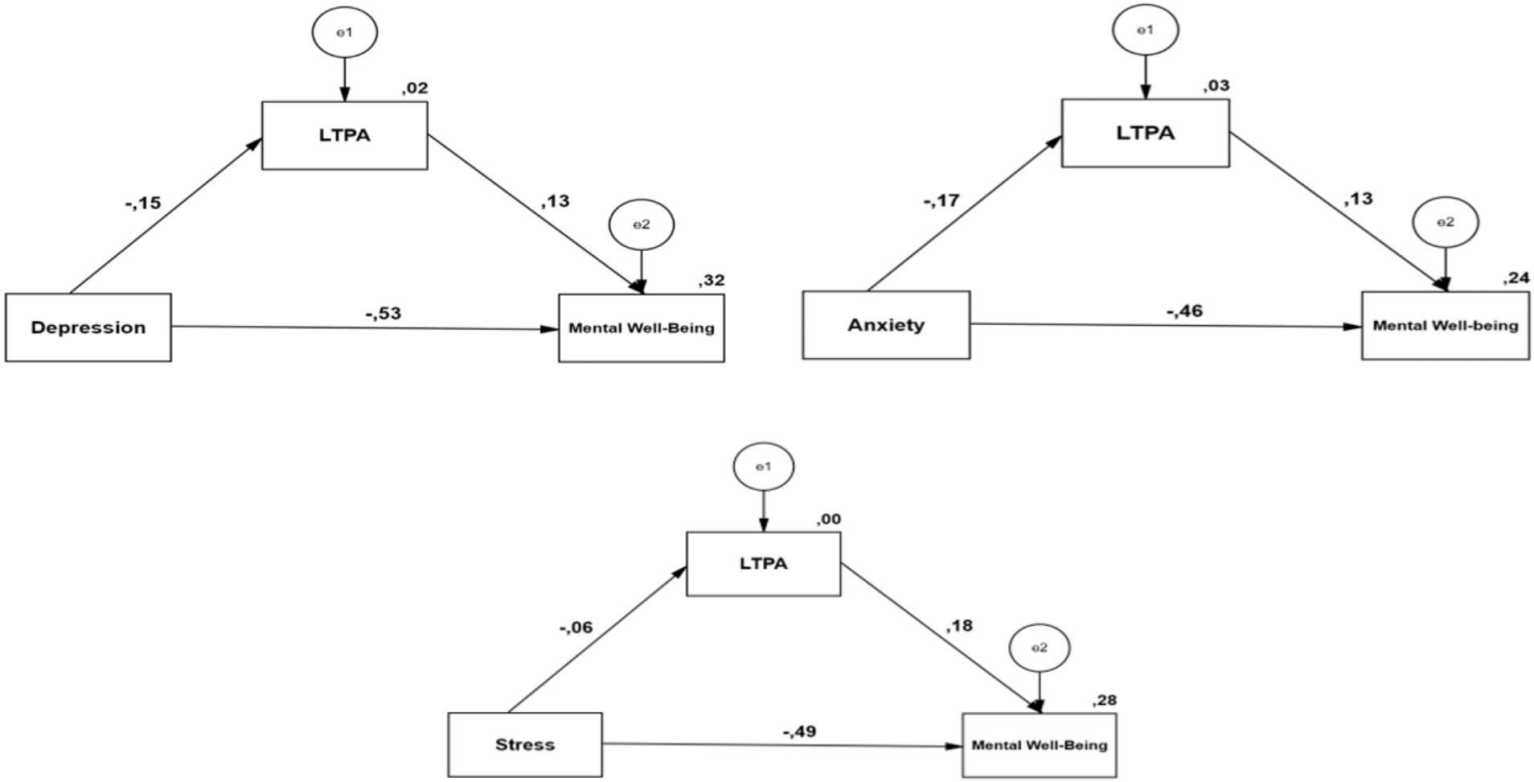
The mediating role of LTPA.

It has been found that men’s high leisure-time physical activity play a protective role between depression and anxiety and mental well-being. However, LTPA did not function as a significant mediator in the relationship between stress and mental well-being (Figure 2). The findings indicate that LTPA were associated with a partial indirect pathway in the relationships between depression/anxiety and mental well-being in men, while no significant indirect pathway was observed for stress. Although LTPA was not associated with a significant indirect pathway in the relationship between stress and mental well-being, higher levels of LTPA were associated with indirect relationships between depression/anxiety and mental well-being. Beyond simply advising men struggling with depression and anxiety in clinical settings to “exercise,” promoting structured and regular participation in leisure-time physical activity may have strategic importance for maintaining mental well-being. The fact that LTPA do not mediate between stress and mental well-being suggests that the effect of LTPA on mental well-being can be explained more by direct neurobiological mechanisms or psychological resources such as self-efficacy. Therefore, future research that separately examines different dimensions of stress (e.g., perceived stress, chronic stress, daily stressors) may contribute to testing the model more precisely.

## Data Availability

Data supporting the findings of this study are available from the corresponding authors upon reasonable request.
